# Latest Overview of the Cyclin-Dependent Kinases 4/6 Inhibitors in Breast Cancer: The Past, the Present and the Future

**DOI:** 10.7150/jca.33079

**Published:** 2019-10-21

**Authors:** Xiu Chen, Di Xu, Xingjiang Li, Jian Zhang, Weilin Xu, Junchen Hou, Wei Zhang, Jinhai Tang

**Affiliations:** 1 Department of General Surgery, the First Affiliated Hospital of Nanjing Medical University, Nanjing, Jiangsu, China.; 2 Changzhou Wujin People's Hospital, Changzhou, Jiangsu, China.

**Keywords:** breast cancer, drug, cyclin-dependent kinases 4/6 inhibitors, adjuvant therapy, neoadjuvant therapy

## Abstract

Endocrine resistance in hormone receptor positive breast cancer patients urges us to develop novel approaches such as inhibitors of the cyclin-dependent kinases (CDK) 4/6 to reverse its resistance. Nowadays, three selective CDK4/6 inhibitors (Palbociclib, Ribociclib and Abemaciclib) are approved by Federal Drug Administration and the European Medicines Agency for the treatment of advanced and metastatic HR+/HER2- breast cancer. However, no consistent conclusion has been reached to its application in other types of breast cancer. Therefore, the purpose of our study was to overview the clinical trials about the beneficial effects of Palbociclib, Ribociclib and Abemaciclib in breast cancer with their tolerable adverse effects, and discuss their resistant mechanisms thus looking for useful biomarkers to predict the efficiency of the CDK4/6 inhibitors. The CDK4/6 inhibitors application after the support of preclinic and clinic data will be helpful to provide other alternatively suitable strategies for different types of breast cancer patients.

## Introduction

For hormone receptor positive (HR+) breast cancer patients, primary or secondary endocrine therapy resistance is an urgent challenge in recent time. Therefore, approaches targeting other pathway small molecules may prevent and delay the endocrine resistance. Indeed, the combination of the steroidal aromatase-inhibitor (Exemestane) and the mTOR-inhibitor (Everolimus) enhances efficacy of endocrine therapy although with increased toxicity. At the same time, inhibitors of the cyclin-dependent kinases (CDK) 4/6 will delay and reverse endocrine resistance with tolerable adverse effects 1, 2.

It is well acknowledged that CDK4/6 inhibitors mainly block retinoblastoma tumor suppressor protein phosphorylation, thus preventing progression through the cell cycle.

For several decades, three drugs of the selective CDK4/6 inhibitors have been approved by the Federal Drug Administration (FDA) and the European Medicines Agency (EMA) for the treatment of advanced and metastatic HR+/HER2- breast cancer. For instance, Palbociclib (PD0332991)^3^ provides no activities against 36 additional kinases and Ribociclib (LEE011) is in lack of activities against CDK1, 2. These two agents are approved for first line treatment in combination with Letrozole. While Palbociclib and Abemaciclib (LY2835219) with attenuated potency against CDK1, 2, 7, 9 are registered for second line therapy with Fulvestrant 4-8.

In spite of numerous completed studies considering the pharmacokinetic activity, safety and efficacy of these three selective CDK4/6 inhibitors, a plenty of ongoing clinical trials are investigating the indispensable position of Palbociclib, Ribociclib and Abemaciclib in different subtypes of breast cancer.

In this review, we mainly overviewed the clinical trials about the beneficial effects of Palbociclib (Table 1), Ribociclib (Table 2) and Abemaciclib (Table 3) in breast cancer with their tolerable adverse effects, and discussed their resistant mechanisms thus looking for useful biomarkers to predict the efficiency of the CDK4/6 inhibitors. Preclinic and clinic data will be helpful to provide alternatively suitable strategies for different types of breast cancer patients.

### Application of CDK4/6 inhibitors in HR+/HER2- advanced breast cancer

Palbociclib, Abemaciclib and Ribociclib all generated significant improvements in median progression free survival (PFS) when combined with an endocrine drug for the hormone receptor-positive advanced breast cancer patients^9^, ^10^.

The Phase III MONALEESA-2 trial (NCT01958021) enrolled 668 postmenopausal HR+/HER2- sub-type breast cancer patients, among them 295 patients were over 65 years old. Randomly, they were randomly grouped to take Ribociclib (600mg/day, 3-weeks-on/1-week-off) as well as Letrozole (2.5 mg/day) as the first-line therapy or placebo and Letrozole until the endpoints. Data summarized that Ribociclib plus Letrozole obviously improved PFS of breast cancer patients, with no different effects between older and younger patients 11, 12. Furthermore, in the US, a three-year data of 263 members showed that Ribociclib plus Letrozole seemed to be an effective and economical approach for HR+/ HER2- advanced breast cancer patients^13^, ^14^.

In the grade II/III HR+/HER2- invasive breast cancer patients, the expression of the Ki67 was dramatically decreased (≥92%) in those receiving combination of Ribociclib and Letrozole comparing to those who receiving Letrozole alone (69%)^15^. An NCT02712723 trial investigated the neoadjuvant use of Ribociclib plus Letrozole. Also, in pretreated HR+/HER2- advanced breast cancer (ABC) patients, after combination of Ribociclib plus Fulvestrant therapy, partial responses (PRs) were observed in patients with prior treatment of Fulvestrant. An ongoing NCT03671330 trial is enrolling 315 Chinese populations to compare the PFS of HR+/ HER2- ABC patients receiving Ribociclib treatment with those who receive placebo.

Fifty early-stage breast cancer patients were enrolled in a phase II neoadjuvant trial. From the results, the complete cell cycle arrest (CCCA: central Ki67≤2.7%) rate was significantly higher in combination of Palbociclib with Anastrozole, compared to Anastrozole monotherapy 16. Recently, the clinical trials PATHWAY, PASIPHAE, INGE-B, NCT02668666, PRECYCLE, NCT03425838 are ongoing to explore the PFS, overall survival (OS), clinical benefit rate (CBR) and quality of life (QoL) in the breast cancer patients.

A cohort of 47 ABC patients with or without PIK3CA mutations who received Abemaciclib therapy, reached a higher disease control rate (CR+PR+SD) in HR+ patients (29 of 36 patients, 81%) than the HR- patients (3 of 9 patients, 33%). Meanwhile, among the HR+ group, the radiographic responses occurred exclusively, the clinical benefit rate (CR+PR+SD≥24 weeks) was 61%, median duration of response was 13.4 months, and median PFS was 8.8 months 17.

A phase I study consisting of 225 ABC or metastatic breast cancer (MBC) patients and a phase II study including 132 patients 18 demonstrated that single-use as well as united-use of Abemaciclib were well effective in HR-positive breast cancer. More intensive studies reported an acceptably safe dose at 200 mg Q12H of Abemaciclib when administered as a single-use in Japanese cancer patients 19.

From the results of several studies, it is easy to conclude the idea that Palbociclib, Abemaciclib and Ribociclib play important roles in the enhancement of survival periods of HR+/HER2- ABC patients. Further trials are ongoing to gather more information and better appropriate dose or combination of CDK4/6 inhibitors in this type of breast cancer.

### Application of CDK4/6 inhibitors in HR+/HER2- metastatic breast cancer

Impressive activities of CDK4/6 inhibitors are demonstrated in ER+/HER2- MBC patients with a great efficacy and low toxicity 20. Simultaneously, MBC patients with a discontinuation of CDK4/6 inhibitors are potential to undergo rapid disease progression 21.

Palbociclib shows great promise in the strategy of HR+ metastatic breast cancer 24. A study of 525 post-menopausal women with HR+/HER2- MBC demonstrated that those who transferred from CDK4/6 inhibitor-based treatment to chemotherapy had more tendency to suffer from recurrent rapidly progressing disease 22.

Vered et al. carried out a Palbociclib Expanded Access Program (EAP) and collected a total of 334 metastatic ER+/HER2- breast cancer patients to prove the good tolerance and the safety of Palbociclib with Letrozole 23.

On the contrary, in face of the twenty-three HR+/HER2- MBC patients with previous use of Everolimus, the adding of Palbociclib-based therapy revealed limited clinical benefits in the PFS, objective response rate (ORR) and CBR 24. Meanwhile, no biomarkers have been detected to define which subtype of patients will benefit from the addition of CDK4/6 inhibitors to endocrine therapy in MBC. In another words, we should not regard the ESR1 mutational status as a biomarker to restrict the use of CDK 4/6 inhibitors 25.

Therefore, more ongoing clinical trials just like PYTHIA, NCT02384239, PACE are conducted to further detect the effectiveness of Palbociclib-based therapy in HR+ metastatic breast cancer.

Ribociclib is reported to express functional Rb protein for its antiproliferative activity in HR+/HER2- metastasis breast cancer 26. Also, NCT03096847, AMICA and RIBBIT trials are discovering the PFS, OS, CBR of Ribociclib in combination with anti-hormonal Therapies.

Besides this, studies about the Abemaciclib used in metastatic breast cancer are numbered the phase II NCT03130439 and the phase III MonarchE clinical trials.

So CDK4/6 inhibitors are potentially effective therapies to promote prognoses of HR+/HER2- metastasis breast cancer patients with further confirmations needed.

### Other types of breast cancer

Activities of CDK4/6 inhibitors in non-luminal breast cancer types were also discussed 27-29.

For HER2+ breast cancer patients, they also gained benefits from the use of Palbociclib, Ribociclib and Abemaciclib. In a reported phase II study, two (5%) of the 37 enrolled patients had HR+/HER2+ breast cancer, without prior HER2-directed treatment. Owing to the single application of Palbociclib, one of them had a partial response, the other had a stable disease lasting for 5 months 3.

Meanwhile, the PATRICIA (NCT02448420) study aims to evaluating the role of Palbociclib when added to the standard Trastuzumab therapy in HER2+ MBC.

Another ongoing phase 2 study (NCT02774681) is evaluating effects of Palbociclib in HR-/HER2+ breast cancer patients with brain metastases. Patients who had received more than two prior chemotherapy regimens, or had uncontrolled neurologic symptoms or leptomeningeal disease will be excluded.

NCT03304080 is discovering whether Palbociclib combined with Anastrozole, Trastuzumab, and Pertuzumab could be a first-line therapy in HR+/HER2+ MBC patients. After the standard four to eight cycles of chemotherapy and anti-HER2 therapy, Palbociclib will be added to the following endocrine therapy in PATINA (NCT02947685) study. The results will give an answer to the investigators if the addition of Palbociclib delays disease progression of HR+/HER2+ MBC patients. A phase 2 study (NCT03054363) is exploring the efficacy of a triple targeted drug combination (Palbociclib, tucatinib and Letrozole) in HR+/HER2+ MBC patients.

A non-randomized, phase I/II study (NCT02657343) will test maximum tolerated dose (MTD), CBR, ORR, PFS, OS and AEs of Ribociclib in combination with Trastuzumab or T-DM1 for advanced or metastatic HER2+ breast cancer patients.

In the phase I study of single Abemaciclib use, eleven HR+/HER2+ MBC patients obtained positive clinical responses. To be more precise, four (36%) patients reached partial response and 7 (64%) patients got stable disease 17. The monarcHER phase II, randomized trial (NCT02675231) will soon come to a conclusion on the PFS, OS, ORR, DoR, CBR and QoL of HR+/HER2+ MBC patients when receiving Abemaciclib and Trastuzumab as well as Fulvestrant.

A Randomized Phase III Trial (PALLAS) is recruiting 5600 participants to compare the invasive disease free survival (iDFS), distant recurrence-free survival (DRFS), locoregional recurrences-free survival (LRRFS) and OS of the collaborative use of Palbociclib with or without other therapies in early breast cancer patients. For the early stage setting of HR+/HER2+ breast cancer patients, the NA-PHER2 (a phase II, neoadjuvant study) evaluated a combination of Palbociclib, Fulvestrant, Trastuzumab and Pertuzumab in order to avoid the use of chemotherapy^30^. In this study, results of 36 patients concluded that the combination therapy had a significant effect on the expression of Ki67 at baseline (p <0.0001). Besides, 27% of patients achieved a pathologic complete response (pCR) before surgery. At the same time, PALTAN (NCT02907918) trial is evaluating the pCR rate of newly diagnosed clinical stage II or III HR+/HER2+ breast cancer patients by the use of Palbociclib in combination with neoadjuvant letrozole and weekly trastuzumab.

Androgen receptor (AR) expression is significantly associated with Rb expression, which is disturbed by CDK4/6 inhibitors. Besides, Rb expression in TNBC was verified to improve tumor sensitivity to Erlotinib and Palbociclib Combination Therapy 31. So as to confirm the role of CKIs in AR+/TNBC breast cancer, stage I-II trials NCT02605486 and BRE15-024 (NCT03090165) are surveying whether Palbociclib in combination with Bicalutamide improve the PFS, CBR of metastatic patients and whether Ribociclib with Bicalutamide promote CBR of TNBC, separately.

In the mentioned phase 2 study, four (11%) of the 37 patients enrolled were TNBC, and all of them rapidly progressed under the Palbociclib treatment 3.

A case report showed that a high-grade primary neuroendocrine breast cancer patient underwent an ideal and durable response to Palbociclib in conjunction with endocrine therapy after non-effectiveness of commonly used platinum-based chemotherapy and endocrine therapy 32.

Although CDK4/6 inhibitors have a great tendency to bring about positive prognosis of HR+/HER2- breast cancer patients, for other type such as HER2+, TNBC or AR+ breast cancer, the application of CDK4/6 inhibitors still needs further intensive studies.

### Adverse effects of CDK4/6 inhibitors

The summary of eight clinical trials illustrates that the first-line or later CDK4/6 inhibitor-based combination therapies bring about the definitely improved progression free survival (PFS), although with inevitable side effects. The most common side effects of the three CDK4/6 inhibitors are neutropenia, leukopenia, fatigue and nausea. Meanwhile, the most common grade 3/4 side effects are neutropenia, leukopenia and diarrhea. Interestingly, the neutropenia initiated by CDK4/6 inhibitors is rather different from chemotherapy-induced neutropenia for its rapid reversibility 33. In detail, Abemaciclib has a 50% lower neutropenia rate in contrast with Palbociclib and Ribociclib according to its better selectivity to CDK4. Therefore, patients receiving Palbociclib will mostly suffer from neutropenia, the Ribociclib from neutropenia, QTc prolongation and hepatobilary toxicity, and the Abemaciclib from gastrointestinal toxicity. Discomforts induced by the CKIs will be recovered rapidly by its reductions or interruptions 34.

Palbociclib was approved to apply in the treatment of advanced breast cancer in February 2015 in the USA. One year Palbociclib-use information post US approval was collected to demonstrate the efficacy of Palbociclib in the real world. Of those, 612 advanced breast cancer patients received Palbociclib concomitantly with Letrozole. Through the complete blood count (CBC) monitoring test during the first cycle of Palbociclib treatment, results came that 74.6% of patients experienced the neutropenia including 47.3% and 8.0% of them with a grade 3 or 4 occurrence, in consistence with the clinical trials PALOMA-2 (56% and 10%) and PALOMA-3 (55% and 11%), respectively^35^.

Moreover, ongoing phase II-IV clinical trials such as PALINA, NCT02600923, NCT02679755 and NCT03633331 are exploring the most frequent adverse effects resulting from the application of Palbociclib in HR+/HER2- advanced breast cancer patients. An ongoing non-randomized, phase I (NCT03065387) study will measure the safety of the pan-HER inhibitor neratinib in combination with Palbociclib in HER2 mutation/amplification advanced breast cancer patients.

The MONALEESA-2 trial also detected the most common adverse effects were neutropenia, leukopenia, and anemia, while elderly patients receiving Ribociclib showed a greater than 10% increase in the incidence of fatigue 11. The phase II clinical trial LEADER and the phase I trial NCT02599363 are under research to deliver more side effects information of Ribociclib treatment in ER+ early breast cancer.

A case reported a 74-year-old breast cancer woman developed the radiation-induced morphea (RIM) after experiencing neoadjuvant CDK4/6 inhibitor Abemaciclib and aromatase inhibitor Anastrozole treatment in combination of the subsequent radiation therapy 36.

In contrast, the toxicity of Abemaciclib was much milder. At the same time, RB S780 phosphorylation inhibition and topoisomerase IIa abundance in skin biopsies as well as in tumor biopsies were newly detected biomarkers to reflect drug responses. Furthermore, reduction of RB phosphorylation by above 60% was predicted for the disease control 17. In the Phase I trial of advanced ER+/HER2- breast cancer, the evaluation of Abemaciclib was performed along with a 72% disease control rate.

Two phase I clinical trials named NCT02057133 and NCT02779751 are studying the adverse effects in the combination of Abemaciclib with endocrine therapy.

### Interactions and combinations with other therapies

All three CDK4/6 inhibitors can be orally taken and their metabolism will be influenced by CYP3A and SULT2A1 enzymes. Therefore, interactions with CYP3A inhibitors or CYP3A inducers may result in an increased toxicity or decreased efficacy of the three CDK4/6 inhibitors, respectively.

Palbociclib was confirmed to be well tolerated when combined with the c-MET inhibitor, Crizotinib, without causing significantly abnormal serum chemistry and hematology levels *in vitro* 37.

It is known that PFKFB3 inhibits ubiquitin proteasome degradation of the heat shock protein90-Cdc37-CDK4 complex to increase CDK4 protein. Researchers found that disrupting the interaction of PFKFB3 and CDK4 by mutating lysine (147) to alanine led to accelerated degradations of CDK4. Therefore, the effect of Palbociclib will be improved by interfering PFKFB3-CDK4 interactions, probably by specific mutant approaches 38.

Triplet joint therapy of Ribociclib with exemestane and Everolimus (mTOR inhibitor), or Letrozole and Alpelisib (PI3Ka-selective inhibitor) were investigated in HR+ ABC patients whose tumor cells endured PI3K/AKT/mTOR and/or cyclin D-CDK4/6-p16-Rb pathway alterations^39^-^41^. Pharmacokinetic indices for Ribociclib and Alpelisib were almost the same with those in their single-agent application. Moreover, after combined use of Ribociclib, the level of Everolimus, metabolized by cytochrome P450 3A4 (CYP3A4), increased by 1.5-to 3-fold, which provided evidence to the possibility of lower doses of Everolimus in combination with Ribociclib, leading to less drug toxicity^42^.

Okada et al. provided a novel antitumor strategy of combining CDK4 inhibitor with autophagy inhibition by either chloroquine (CQ) or knockdown of ATG5 or BECN1 to induce synthetic lethal toxicity for BT474, MDA-MB435S, SKBr3 breast cancer cells 43.

The impact of Abemaciclib on tumor immune microenvironment was investigated by *in vitro* and *in vivo* assays. Abemaciclib was reported to activate T cell inflammatory functions and antigen presentation genes in tumors. So a combination of Abemaciclib and anti-PD-L1 therapy, that modulate T cell anti-tumor immunity, was proven to delay of tumor growth 44.

The combination of Palbociclib, Ribociclib or Abemaciclib with other pathway molecule drugs is helpful to boost the therapy responses with decreased drug doses and tolerable toxicities.

### Resistance and solutions

After continual application of CDK4/6 inhibitors, the unwanted acquired resistance is emerging. Therefore, we need to find potential post-CDK4/6 inhibitor therapeutic strategies to cope with its resistance 45.

Studies have reported that ESR1 mutation with ongoing trial's confirmation (NCT02738866) and loss of ER, upregulation of fibroblast growth factor receptor (FGFR) amplification, and acquired RB1 mutations 46 were in correlation with acquired CDK4/6 inhibitors resistance.

A clinical report compared the circulating tumor DNA (ctDNA) pre-exposure and after-exposure to CDK4/6 inhibitor (Palbociclib or Ribociclib) in three metastatic BC patients, discovering the substitution in donor splicing site of exon 8 of the RB1 gene, substitution in donor splicing site of exon 22 together with exon 19 deletion and exon 3 insertion, mutation in exon 16 H483Y, respectively 46.

Combination of phosphatidylinositol-3 kinase (PI3K) or mammalian target of rapamycin (mTOR) inhibitors with CDK4/6 inhibitors and endocrine inhibitors has a tendency to prolong CDK4/6 inhibitor sensitivity 47.

#### Palbociclib

By using an ATP/ADP probe-based chemoproteomics platform, Nomanbhoy et al were able to predict whether a patient was resistant to palbociclib or not 48.

The efficacy of Palbociclib is limited by increased CDK2 and RBL1 expression 49. In details, CCNE1 amplification upregulated levels of CDK2/CCNE1, following by RB inactivation and CDK4/6 inhibitors resistance 50. Studies described synergy between CDK4 and PI3K inhibitors, for PI3K inhibitors would suppress cyclin D1 expression, leading to impediments to the presence of Palbociclib-resistant clones 51. A preclinical study demonstrated that mechanisms of resistance to CDK4/6 inhibitors differ between Palbociclib and Ribociclib. In this study, Palbociclib-resistant cells presented increased cycle E protein levels while Ribociclib-resistant clones produced increased E2F1^52^. The consistent discovery was uncovered that resistance to Palbociclib was associated with non-luminal subtypes as well as persistent E2F-target gene expression 16.

The presence of T172-phosphorylated CDK4 was proved to predict the sensitivity to Palbociclib in breast cancer, which could help to select a subset of drug sensitive patients in case of inefficacy 53.

The ongoing phase IV NCT03401359 trial plans to recruit 100 metastatic breast cancer patients to discover biomarkers of acquired resistance response to Palbociclib from whole exome sequencing, RNASeq, circulating tumor DNA and flow cytometry approaches, thus making the targeted treatments more effective and flexible.

#### Ribociclib

The transport of Ribociclib is affected by ABCB1 across the membranes and it has potential to deliver drug-drug interactions (DDIs) via ABCB1 and ABCG2 transporters as well as CYP isoforms, probably leading to beneficial MDR-reversing 54.

Risi and colleagues calculated from ten studies to detected a potentially predictive biomarker namely RBsig, a gene signature of RB loss. They found that low expression of RBsig separated a subset of ER+/HER2+ breast cancer patients with low pCR rates as they were resistant to neoadjuvant chemotherapy, anti-HER2 therapy and CDK4/6 inhibitor combinations 55.

Meanwhile, researchers were challenged to discover approaches to overcome the resistance to CDK4/6 inhibitors. PACE trial (NCT03147287) and MAINTAIN trial (NCT02632045) are ongoing to evaluate the efficacy of Ribociclib in HR+ MBC patients who have received CDK4/6 inhibitor before. TRINITI-1 trial (NCT01857193) explores the effects of triplet combination of Everolimus with Ribociclib and Exemestane-progressed patients who had prior use of CDK4/6 inhibitor. Also, NCT03238196 studies triplet combination of Erdafitinib with Ribociclib and Fulvestrant for patients with HR+ MBC 56.

The PI3K/AKT/mTOR pathway is well known as a critical pathway in HR positive breast cancer. So combination of Ribociclib with the PI3K inhibitor alpelisib (BYL719) brought about enhanced tumor regression, response rates and PFS versus single-agent treatment 57-59. But recent data indicated that the application of PI3K inhibitors seems disappointing because of its modest effects and great toxicities, while the mTOR inhibitor everolimus evidently improves PFS when added to ET with less toxicity 60. Also, recent data shows that CDK4/6 inhibitor-resistant breast cancer cell lines tend to reactivate the CDK-RB-E2F pathway, but are sensitive to the mTORC1/2 inhibitors 61. Therefore, combination of an mTOR or mTORC1/2 inhibitor with a CDK4/6 inhibitor will produce more effects on E2F-dependent transcription as well as regressions of cell growths, which overcome the resistance to the sole use of CDK4/6 inhibitors and delay initiation of chemotherapy.

#### Abemaciclib

TP53 mutations may be a potential biomarker to predict an Abemaciclib-nonresponding type of breast cancer patients 17.

In brief, more effective biomarkers for CDK4/6 inhibitors resistant predictors are needed to select the best available treatment and generate better prognosis in the rather poor type of breast cancer patients.

## Discussion

In the case of HR+/HER2- breast cancer, endocrine therapy is usually the first-line therapy depending on the menopausal status of the patient. However, acquired resistance to endocrine treatments is in desperate need of effective solutions. Therefore several alternate choices including high-dose Fulvestrant or Everolimus, PI3K inhibitors, Entinostat or CDK 4/6 inhibitors are under research 62, 63.

Multiple evidences have held the critical role of CDK-RB-E2F pathway in cell proliferation of breast cancer 64. Preclinical evidence demonstrated that the interruption of cyclin D1/CDK4/6/Rb axis in breast cancer encountered the disruption of cell cycle. The addition of CDK4/6 inhibitors to endocrine therapy have produced increased efficacy and improved PFS. And three CDK4/6 inhibitors (Palbociclib, Ribociclib and Abemaciclib) have been approved by the US Food and Drug Administration 65-68. The three CDK inhibitors bind to CDKs family and interrupt the binding of ATP. They form hydrogen bonds with CDKs and interact with CS6 and CS7 on the catalytic spine, playing a significant role in the regulation of cell cycle 69.

Palbociclib (PD0332991) is the first oral CDK4/6 inhibitor with a simultaneous use of letrozole as first-line therapy, resulting in the significantly improved median PFS in advanced breast cancer with ER positive and HER2 negative (24.8 vs 14.5 months [PALOMA2]) as well as with Fulvestrant in endocrine pretreated patients (9.2 vs 3.8 months [PALOMA-3])^70^, ^71^.

MONARCH 1, 2, and 3 clinical trials established Abemaciclib as an orally effective CDK4/6 inhibitor for HR+/HER2- advanced or metastatic breast cancer 72, 73.

Up to now, researchers have adopted sequential administration of the pan-CDK inhibitor Roscovitine following by doxorubicin to TNBC cells, resulting in increased DNA damages, reduced recruitment of homologous recombination proteins with decreased tumor size, improved overall survival in comparison with single or concomitant treatment in TNBC 74. Therefore, the application of CDK4/6 inhibitors might also produce similar prognosis in TNBC and other subtype of breast cancer while several ongoing studies are under implementation.

With splendid advantages of the addition use of CDK4/6 inhibitors, adverse effects are unwelcomed but inevitable. For example, Palbociclib mainly causes bone marrow suppression however it is always reversible by absence or withdrawal of Palbociclib, clearly differentiated from the apoptotic cell death caused by chemotherapy 75.

Recently, radiotherapy plays a more and more vital role in the comprehensive treatment of breast cancer patients 76. Besides, latest literature has discussed the effect of Ribociclib in combination with radiotherapy in head and neck squamous cell carcinoma. Results suggested that Ribociclib was a potential radiosensitizer by enhancing the cytotoxic effects of radiation in OML1 cells and reversing the radiation resistance in OML1-resistent cells. So evidences on Ribociclib during radiotherapy for breast cancer cells should be collected and taken into consideration 77.

Interestingly, CDK4/6 inhibitors might display influences on the tumor microenvironment. For instance, inhibition of CDK4 activity would significantly reduce the stabilization of autophagy-mediated proteasome NOXA and induce cell apoptosis in mantle cell lymphoma 78. Also, studies demonstrated that cyclin D-CDK4 kinase destabilized PD-L1 via Cullin3^SPOP^ to control cancer immune surveillance and revealed the potential combination of CDK4/6 inhibitors and PD-1/PD-L1 blockade to enhance therapeutic efficacy for human cancers 79. Simultaneously, combined targeting of MDM2 and CDK4 was synergistic in dedifferentiated liposarcomas. The use of RG7388 and Palbociclib together was found to exert a greater anti-tumor effect than either drugs alone 80. New findings will be helpful to emphasize the prevalent action of CDK4/6 inhibitors throughout the treatment process in breast cancer patients.

Overall we skimmed researches concerning the efficacy and safety of Palbociclib, Ribociclib and Abemaciclib in diverse types of breast cancer patients. Although CDK4/6 inhibitors generate side effects especially hematologic changes, their distinct superiority is more glaring in breast cancer treatments. So with inevitable resistant mechanisms, more and more clinical trials are seeking for potential biomarkers to predict the validity of the CDK4/6 inhibitors, thus bringing in more optionally effective therapy strategies to breast cancer patients, even to the currently incurable breast cancer patients.

## Figures and Tables

**Table 1:** Studies of Palbociclib

Study name/ID	Phase	Subtype	Prior treatment	Allocation	Number	Combination drugs	Aims
PATHWAY/ NCT03423199	3	HR+/HER2- ABC	N/A	Non-Randomized	180	Tamoxifen ± Goserelin	PFS,OS
PASIPHAE/ NCT03322215	2	HR+/HER2- ABC	endocrine	Randomized	196	Fulvestrant	PFS,OS
INGE-B/ NCT02894398	2	HR+/HER2- ABC	N/A	Non-Randomized	360	none	CBR
NCT02668666	2	HR+/HER2- ABC	N/A	Non-Randomized	71	Tamoxifen	Response Rates,PFS,CBR,OS
PRECYCLE/ NCT03220178	4	HR+/HER2- ABC	N/A	Non-Randomized	960	none	DQoL,PFS, OS
NCT03425838	3	HR+/HER2- ABC	N/A	Non-Randomized	1050	AI or Fulvestrant	PFS2,OS
PYTHIA/ NCT02536742	2	HR+/HER2- MBC	endocrine	Non-Randomized	120	Fulvestrant	PFS
NCT02384239	2	HR+/HER2- MBC	N/A	Non-Randomized	70	none	Tumor Progression,PFS
PACE/ NCT03147287	2	HR+/HER2- MBC	endocrine	Randomized	220	Fulvestrant and Avelumab or Fulvestrant	PFS,OSR
PATRICIA/ NCT02448420	2	HR+/HER2+ MBC	N/A	Non-Randomized	138	None	PFS
PATINA/ NCT02947685	3	HR+/HER2+ MBC	N/A	Non-Randomized	496	None	PFS,OS
PALLAS/ NCT02513394	3	HR+/HER2- EBC	neo/adjuvant therapy	Randomized	5600	standard adjuvant endocrine therapy	iDFS,DRFS,LRRFS,OS
NCT02605486	1,2	AR+ MBC	endocrine or chemotherapy	Non-Randomized	51	Bicalutamide	PFS,Toxicity assessment
BRE15-024/ NCT03090165	1,2	AR+ TNBC	N/A	Non-Randomized	58	Bicalutamide	CBR,ORR
NCT02600923	3	HR+/HER2- ABC	none	Non-Randomized	130	Letrozole	AE
NCT02679755	4	HR+/HER2- ABC	none	Non-Randomized	300	Letrozole	AE
NCT02626507	1	ER+/HER2- BC	none	Non-Randomized	18	None	AE
PALINA/ NCT02692755	2,3	HR+/HER2- BC	N/A	Non-Randomized	35	Letrozole or Fulvestrant	AE
NCT03401359	N/A	HR+ MBC	N/A	Non-Randomized	100	Endocrine	Biomarker of acquired resistance
NCT03238196	1b	ER+/HER2-/FGFR-amplified MBC	N/A	Non-Randomized	32	Fulvestrant and Erdafitinib	AE,DLT,MTD,Next Generation Sequencing
NCT02774681	2	HR-/HER2+ MBC	N/A	Single group	12	Trastuzumab	Radiographic response rate in the CNS,AE,OS,PFS,ORR
NCT03304080	1/2	HR+/HER2+ MBC	N/A	Single group	36	Anastrozole, Trastuzumab, Pertuzumab	DLT,MTD,CBR,PFS,AE
NCT03065387	1	HER2 Mutation or Amplification	N/A	Non-Randomized	120	Neratinib	MTD
NCT03054363	1b/2	HR+/HER2+ MBC		Single group	25	Tucatinib, Letrozole	AE,PFS
PALTAN/ NCT02907918	2	Stage II-III ER+/HER2+ BC	none	Single group	48	Letrozole, Trastuzumab, or Goserelin	Response Rates, AE, Outcomes

CBR: Clinical Benefit Rate; PFS: Progression Free Survival; iDFS: Invasive Disease Free Survival; DRFS: Distant Recurrence-free Survival; LRRFS: Locoregional Recurrences-free Survival; OS: Overall Survival; ORR: Objective Response Rate; PFS2: Progression-free survival after two lines of treatment; PFS6: Progression-Free Survival at 6 months; DLT: dose limiting toxicity; MTD: Maximum Tolerated Dose.

**Table 2:** Studies of Ribociclib

Study name/ID	Phase	Subtype	Prior treatment	Allocation	Number	Combination drugs	Aims
NCT03671330	2	HR+/HER2- ABC	N/A	Randomized	315	none	PFS
MONALEESA-2/ NCT01958021	3	HR+/HER2- ABC	none	Randomized	670	Letrozole	PFS,OS,ORR,CBR
NCT02712723	2	HR+/HER2- ABC	N/A	Randomized	120	Letrozole	PEPI rate, pCR
NCT03096847	3	HR+/HER2- MBC	N/A	Non-Randomized	500	None	CBR,PFS
AMICA/ NCT03555877	2	HR+/HER2- MBC	N/A	Non-Randomized	150	None	PFS,OS,CBR
RIBBIT/ NCT03462251	3	HR+/HER2- MBC	N/A	Non-Randomized	160	None	PFS,OS,CBR
LEADER/ NCT03285412	2	ER+ EBC	N/A	Non-Randomized	120	Endocrine	AE, DFS
NCT02599363	1	Rb+ ABC	N/A	Non-Randomized	28	None	AE
MAINTAIN/ NCT02632045	2	HR+ HER2- ABC	N/A	Randomized	132	Fulvestrant	ORR
TRINITI-1 trial/ NCT01857193	1	ER+/HER2- ABC	N/A	Randomized	132	Everolimus and Exemestane or Exemestane	DLT,safety and tolerability
NCT02657343	1b/2	HER2+ ABC/MBC	N/A	Non-Randomized	86	T-DM1 or Trastuzumab or Fulvestrant	MTD,ORR,PFS,OS

CBR: Clinical Benefit Rate; PFS: Progression Free Survival; iDFS: Invasive Disease Free Survival; DRFS: Distant Recurrence-free Survival; LRRFS: Locoregional Recurrences-free Survival; OS: Overall Survival; ORR: Objective Response Rate; DLT: dose limiting toxicity; PEPI: Pre-operative Endocrine Prognostic Index.

**Table 3:** Studies of Abemaciclib

Study name/ID	Phase	Subtype	Prior treatment	Allocation	Number	Combination drugs	Aims
NCT03130439	2	Rb+ TNMBC	N/A	Non-Randomized	37	N/A	ORR,PFS,OS,DFS
monarchE/NCT03155997	3	HR+/HER2- MBC	N/A	Randomized	4580	standard adjuvant endocrine therapy	IDFS
NCT02057133	1	BC	N/A	Non-Randomized	198	N/A	AE
NCT02779751	1	BC	N/A	Non-Randomized	100	N/A	AE
monarcHER/NCT02675231	2	HR+/HER2+ MBC		Randomized	225	Trastuzumab, Fulvestrant	PFS,OS

PFS: Progression Free Survival; IDFS: Invasive Disease Free Survival; OS: Overall Survival; ORR: Objective Response Rate
